# Beyond the Disc: Positional Differences in Morphological and Physical Performance Characteristics Among Male Ultimate Frisbee Players

**DOI:** 10.3390/jfmk11010128

**Published:** 2026-03-22

**Authors:** Cristian Hernández, María Alejandra Camacho-Villa, Nuria Sánchez-Hernández, Luis Gabriel Rangel Caballero, Jorge Gómez-Camacho, Juan Carlos Saavedra, Jorge Enrique Buitrago-Espitia, Adrián De la Rosa

**Affiliations:** 1 Programa de Maestría en Ciencias de Deporte, Universidad Santo Tomás, Bucaramanga 681011, Colombia; cristian.hernandez01@ustabuca.edu.co (C.H.); jorge.gomez09@ustabuca.edu.co (J.G.-C.); 2 Pain Study Group (GED), Physical Therapy School, Universidad Industrial de Santander, Bucaramanga 680002, Colombia; maria.camacho3@correo.uis.edu.co; 3 Performance and Health Group, Department of Physical Education and Sport, Faculty of Sports Sciences and Physical Education, University of A Coruna, 15001 A Coruña, Spain; 4 Physical Education and Sports Department, University of Valencia, 46010 Valencia, Spain; nuria.e.sanchez@uv.es; 5 Grupo Ser, Cultura y Movimiento, Facultad de Cultura Física Deporte y Recreación, Universidad Santo Tomás, Bucaramanga 681011, Colombia; dcultu@ustabuca.edu.co; 6 Physical Activity and Sport Program, Unidades Tecnológicas, Bucaramanga 680006, Colombia; juancs19@hotmail.com; 7 Body, Physical Activity and Sport Study Group (GECAFD), Sports Department, Universidad Industrial de Santander, Bucaramanga 680002, Colombia; joebuies@uis.edu.co; 8 Freshage Research Group, Department of Physiology, Faculty of Medicine, University of Valencia, CIBERFES, Fundación Investigación Hospital Clínico Universitario/INCLIVA, 46010 Valencia, Spain

**Keywords:** team sports, overhead sport, physical performance, flying disc

## Abstract

**Background:** Ultimate Frisbee (UF) is an intermittent team sport with distinct positional roles (cutters and handlers), yet evidence integrating anthropometric, body composition, and physical performance profiles by playing position remains limited. This study aimed to examine positional differences in these variables among male UF players. **Methods:** Forty male players (age: 25.13 ± 3.76 years; 7.0 ± 2.5 years of training experience) participated in this cross-sectional design, including 20 cutters and 20 handlers. Anthropometry, body composition, and dynamic balance variables were analyzed using independent-samples *t*-tests or Mann–Whitney U tests, as appropriate. Positional differences in somatotype and physical performance were analyzed using a one-way multivariate analysis of variance (MANOVA). **Results:** No positional differences were observed in general anthropometric variables (*p* > 0.05). However, handlers exhibited higher body fat percentage (14.32 ± 2.37 vs. 11.95 ± 2.45; *p* = 0.028), fat mass (11.08 ± 2.51 vs. 8.95 ± 2.67 kg; *p* = 0.049), and endomorphy (4.15 ± 1.22 vs. 2.99 ± 1.30; *p* = 0.002) than cutters. In contrast, cutters demonstrated higher speed (20 m sprint: 3.11 ± 0.17 vs. 3.21 ± 0.15 s; *p* < 0.05), agility (10.16 ± 0.69 vs. 10.69 ± 0.61 s; *p* < 0.05), and vertical jump performance (Counter Movement Jump: 40.93 ± 6.54 vs. 36.38 ± 4.71 cm; *p* < 0.05; Abalakov: 46.39 ± 7.88 vs. 40.20 ± 4.68 cm; *p* < 0.01). No differences were found in intermittent endurance (Yo-Yo Intermitent Recovery Test1): 982 ± 354 vs. 940 ± 348 m), upper-limb power, or dynamic balance. **Conclusions:** These findings indicate that playing position in UF is characterized by distinct body composition and lower-limb neuromuscular performance profiles, whereas intermittent endurance, upper-limb power, and balance represent shared physical requirements across positions.

## 1. Introduction

Ultimate Frisbee (UF) is a fast-growing, non-contact sport that has expanded substantially worldwide in recent decades, engaging millions of players across more than 50 countries [1]. The game is played by two seven-player teams on a grass field with end zones, with scoring achieved by catching the disc in the opponent’s end zone [1]. A defining characteristic of UF is that players are not allowed to run while in possession of the disc, which emphasizes off-the-disc movement, spatial awareness, and coordinated team play. These characteristics impose complex physical and physiological demands that differentiate UF from field-based team sports [2].

The physiological demands of UF align with those of intermittent sports, characterized by repeated bouts of high-intensity activity alternated with periods of low intensity or passive recovery [3,4]. These intermittent demands place importance on aerobic endurance, which supports repeated high-intensity efforts and recovery throughout play, as previously reported [4]. During a match, athletes perform frequent accelerations, decelerations, changes in direction (COD), jumps, and short sprints, combined with low-intensity actions. Previous studies have shown that ultimate frisbee players (UFP) typically cover approximately 4.7 km per game, with nearly 20% of this distance covered at high intensity [4]. Thus, these physical requirements place considerable stress on the neuromuscular system, particularly on the lower limbs.

Consequently, physical qualities such as speed, agility, and lower-limb power are widely considered key contributors to performance in UF [5]. Speed facilitates separation from defenders and effective defensive tracking, while agility underpins rapid COD in response to opponents’ movements and dynamic game situations [5,6,7]. Additionally, lower-limb power, commonly assessed through vertical jump performance, is relevant given the frequent need to jump for disc reception or defensive blocks.

However, the extent to which these physiological and neuromuscular demands are distributed among players may depend on tactical role during match play [5,7,8]. Players are commonly categorized as cutters or handlers based on their primary offensive and defensive responsibilities [8]. Cutters primarily create space through rapid off-the-disc movements and end-zone attacks, which may emphasize short sprints, acceleration, and explosive actions. In contrast, handlers typically facilitate disc movement across the field, requiring frequent involvement in passing sequences, positional adjustments, and decision-making under pressure [1,9].

Regarding physical performance, these tactical roles are likely associated with distinct demand profiles. Cutters are expected to rely more on repeated-sprint ability, acceleration, change-of-direction speed, and lower-limb power to generate separation and attack space [8]. Conversely, handlers may be characterized by greater involvement in submaximal movements, postural control, and upper-limb function to sustain disc circulation and decision-making under pressure.

Although these functional distinctions suggest potential differences in physical demands, it remains unclear which specific performance variables meaningfully differentiate playing positions in UF, as empirical evidence on positional physical profiles is limited [5,7]. These playing position requirements are likely influenced by individual anthropometric and body composition profiles, which are recognized contributors to performance in intermittent team sports (i.e., sprint capacity, power production, and movement efficiency) [10,11]. In team sports like UF, body composition characteristics may influence physical performance by affecting speed, acceleration, and change-of-direction ability [12]. Skinfold thickness assessment is widely used to estimate subcutaneous adiposity in athletes, and reporting not only body fat %, but also skinfold sums, facilitates comparisons across studies employing different anthropometric protocols [13,14,15].

In the context of UF, players have been described as predominantly mesomorphic in somatotype, reflecting a balance of muscular development and low-fat mass suited to the sport’s high-intensity, multidirectional demands [16]. Somatotype, defined by the relative predominance of endomorphy (adiposity), mesomorphy (musculoskeletal robustness), and ectomorphy (linearity), provides a standardized method to characterize body morphology. Variation across these components may influence sprint capacity, power production, and movement efficiency in intermittent team sports [17]. However, evidence linking somatotype to playing position in UF remains scarce and inconclusive, limiting understanding of whether morphological attributes meaningfully differentiate positional roles.

Beyond morphological characteristics, performance in UF also depends on sport-specific technical actions that place distinct demands on upper-limb function and postural control [18]. Throwing and catching the disc rely on effective hand–disc interactions, in which grip strength and upper-extremity power contribute to disc control, throwing accuracy, and injury prevention [18,19].

Despite the centrality of these actions to gameplay, limited research has examined handgrip strength (HGS) or upper-limb power in UFP. Similarly, dynamic balance likely plays an essential role in maintaining postural stability during cutting maneuvers, defensive marking, and aerial actions, as described in other sports [20,21,22]. Nevertheless, balance-related performance has received little attention in the UF literature. Thus, despite UF’s increasing popularity, comprehensive evidence integrating anthropometric characteristics, body composition, and multiple performance domains remains limited.

In particular, few studies have examined these attributes simultaneously while considering playing position, making it difficult to determine which physical characteristics meaningfully differentiate positional roles and which represent shared performance requirements across players [5,23,24]. Addressing this gap is essential to advancing position-specific profiling, supporting evidence-based training prescriptions, and improving talent identification strategies at UF.

Therefore, the present study aimed to examine positional differences in anthropometric characteristics, body composition, and physical performance among male UF players. We hypothesized that significant differences in anthropometric, body composition, somatotype, and physical performance variables would be observed across positions.

## 2. Materials and Methods

### 2.1. Design and Participants

The present study employed a cross-sectional analytical design with convenience sampling and was conducted in Bucaramanga, Colombia, between July and August 2025.

Forty UFP (men) participated in the study (age: 25.13 ± 3.76 years; weight 75.35 ± 7.88 kg; height 175.50 ± 5.83 cm; 7.0 ± 2.50 years of training experience). All participants were competitive club-level athletes affiliated with officially recognized clubs of the Liga Santandereana de Disco Volador. They regularly competed in federation-recognized regional and national events within the club-based competitive structure of UF. Players were grouped by playing position into handlers (n = 20) and cutters (n = 20).

No formal a priori sample size calculation was performed, as the study used a convenience sample comprising all eligible players available at the time of data collection. A sensitivity analysis indicated that the final sample size (n = 40) provided adequate power (80%) to detect large between-position effects, whereas small to moderate effects may have remained undetected.

All participants were informed about the study, including its risks and benefits. If, after this explanation, they chose not to be included in the analysis, this decision did not adversely affect their current or future team selection. All athletes included provided written informed consent for testing and data collection. The research complied with the Helsinki Declaration, and the protocol was approved by the Ethics Committee for Human Beings at the Universidad Santo Tomás (Approval No. 06062025; 6 June 2025).

### 2.2. Selection Criteria

The inclusion criteria were as follows: (i) being at least 18 years old; (ii) being a member of a club with sporting recognition endorsed by the Liga Santandereana de Disco Volador (LSDV), and regularly participating in structured training and official competitive events at the regional level; (iii) being free of neuromuscular, orthopedic, or neurological conditions that might interfere with the physical performance assessment; and (iv) training at least three times a week.

Exclusion criteria included: (i) any acute or recent musculoskeletal injury or pain that limited training or performance within the previous 4–6 weeks; (ii) surgery within the previous 6–12 months or incomplete rehabilitation; (iii) inability to complete the full testing battery or noncompliance with pre-test standardization.

### 2.3. Testing Procedures

Data were collected at the sports facilities of the private educational institution Pio X. Physical performance variables were assessed on the school soccer field, while anthropometric measurements were taken in a private room to ensure participants’ privacy.

**Figure 1:**
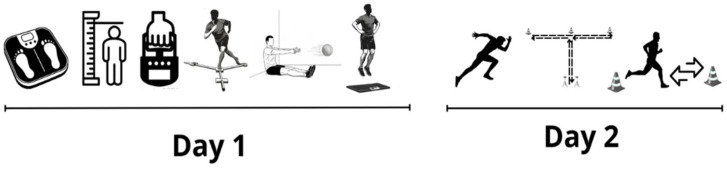
Schematic representation of the assessment order across the two testing days. Day 1 included anthropometric measurements (body weight and height), somatotype assessment, HGS, dynamic balance, upper-limb power, and vertical jump performance. Day 2, conducted 48 h later, included linear sprint testing, agility assessment, and intermittent endurance evaluation. The schematic illustrates the sequential order of testing procedures.

Data were collected in the afternoon (between 2:00 and 4:00 p.m.). During the previous 24 h, participants were not required to perform physical activity or training with their club and were instructed to refrain from consuming caffeine-containing beverages (e.g., coffee, tea, caffeinated soft drinks), energy drinks, and pre-workout supplements during the 24 h preceding each testing session. They were also asked to maintain their habitual hydration practices; however, hydration status was not objectively assessed prior to testing. Evaluations were conducted in the following order: On the first day, anthropometric measurements, HGS, dynamic balance, upper-limb power, and lower-limb power were assessed. The next day, after 48 h, speed, agility, and endurance were assessed. Assessments were conducted during the competitive period (Figure 1).

#### 2.3.1. Anthropometric Measurements and Body Composition

The variables were measured by a professional certified by the International Society for the Advancement of Kinanthropometry (ISAK) as a level 2. Body weight was measured to the nearest 0.1 kg using a bioelectrical impedance analysis device (TANITA BC 240 MA, Tanita Corporation, Arlington Heights, IL, USA). Height and sitting height were measured to the nearest 0.1 cm using a mechanical stadiometer platform (Seca^®^ 274, Hamburg, Germany; TEM = 0.019%) and a 30  ×  40  ×  50 cm high wooden anthropometric box. In addition, the wingspan was measured with a flexible segmometer (Cerscorf; Porto, Portugal).

The following skinfolds were measured: biceps, triceps, iliac crest, suprailiac, subscapular, abdominal, calf, and thigh. Measurements were taken with a plicometer (Cescorf, Porto Alegre, Brazil). The body circumferences considered in the study were: relaxed arm, flexed arm, forearm, thorax, minimum waist, hips, thigh, and maximum calf. Measurements were taken using a tape measure (Cescorf, Porto Alegre, Brazil; measurement range up to 100 cm; accuracy 0.1 cm). Additional arm and hand dimensions, such as arm length, forearm length, hand length, and first–fifth finger distance, were also measured. The bone component was assessed with an anthropometer (Cescorf, Porto Alegre, Brazil), and the bicondylar width of the femur and humerus was considered. The Heath–Carter method was used to calculate anthropometric somatotypes [25,26]:

**Endomorphy** = −0.7182 + 0.1451 × (X) − 0.00068 × (X)^2^ + 0.0000014 × (X)^3^.

where X = (sum of triceps, subscapular, and supraspinal skinfolds in mm) × (170.18/height (cm)).

The mesomorphy component was calculated using the following equation:

**Mesomorphy** = (0.858 × humerus breadth) + (0.601 × femur breadth) + (0.188 × corrected flexed arm girth) + (0.161 × corrected calf girth) − (0.131 × height (cm)) + 4.5

The **ectomorphy** component was calculated using the height–weight ratio (HWR), defined as the ratio of height (cm) to the cube root of body weight (kg). The classification of ectomorphy was established according to the following criteria:

If HWR ≥ 40.75, then Ectomorphy = (0.732 × HWR) − 28.58
  If HWR > 38.25 and <40.75, then Ectomorphy = (0.463 × HWR) − 17.63
  If HWR ≤ 38.25, then Ectomorphy = 0.1 [26]

where

HWR = height/weight^1/3^.

The X and Y axes are used for the somatochart, calculated using the following equations:

X = ectomorphy component − endomorphy component

Y = 2 × mesomorphy component − (endomorphy component + ectomorphy component)

Based on the measurements, body composition was estimated using the calculations proposed by De Rose and Guimaraes [27], based on their four-compartment model (fat mass, bone mass, muscle mass, and residual mass). BF% was calculated using the Faulkner equation; fat mass was calculated as the product of BF% and body mass, divided by 100; bone mass from the bicondylar breadths of the humerus and femur [28]; residual mass as a fixed proportion of body mass [29]; and muscle mass was calculated by subtracting the sum of fat mass, bone mass, and residual mass from total body mass.

The sum of six skinfolds (triceps, subscapular, supraspinal, abdominal, front thigh, and medial calf) and the sum of eight skinfolds (triceps, biceps, subscapular, supraspinal, iliac crest, abdominal, front thigh, and medial calf) were also calculated, as commonly used indicator of body fatness [13,30]. In addition, the upper arm muscle area was estimated bilaterally using the equation proposed by Frisancho [31].

#### 2.3.2. Handgrip Strength Test

HGS was measured with a portable digital hand dynamometer (Takei 5401; Tokyo, Japan). The athlete stood with the arm at the side, the elbow flexed at 90°, and the forearm in neutral rotation. The wrist was also kept in a neutral position, aligned with the forearm holding the instrument, which was adjusted to each athlete’s hand to ensure effective testing. Athletes performed three 5 s maximal efforts with each hand, with 60 s rests between attempts. The order of testing between hands was randomized to minimize potential order effects, and the highest value from each hand was used for subsequent analysis [32,33].

#### 2.3.3. Seated Medicine Ball Throw Test

**Figure 2:**
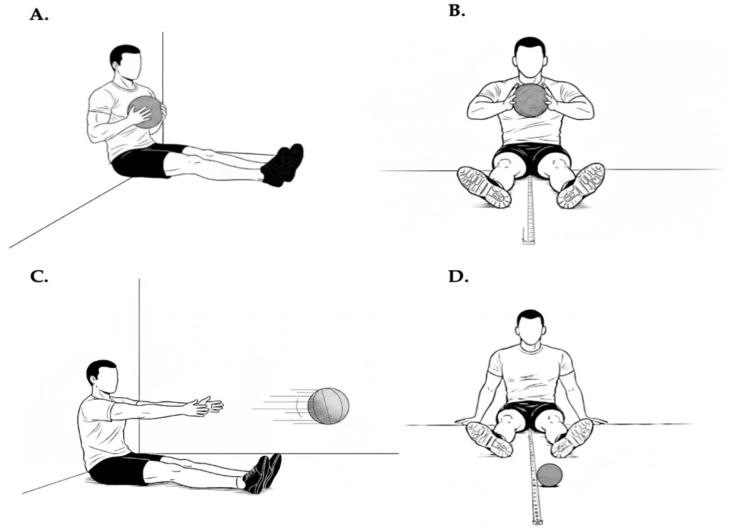
Set up of the seated medicine ball throw test. The sequence shows the standardized starting position with trunk support (**A**,**B**), the throwing action and ball release (**C**), and the measurement of the horizontal distance achieved (**D**), used as an indicator of upper-limb power.

The Seated Medicine Ball Throw (SMBT) was used to assess upper-limb power, as described by Borms & Cools [34]. Briefly, participants sat with their legs extended and their backs, shoulders, and heads in contact with the wall. They held a 2 kg medicine ball in both hands, which was coated with chalk to ensure it left a mark when it hit the ground (Figure 2). Elbows were flexed, and shoulders were abducted to 90 degrees.

The athlete threw the ball as far forward as possible in a straight line, without losing contact with the back, head, and shoulders of the wall during the throw. A 10 m tape measure was placed on the floor to evaluate the distance of the throw. Each athlete completed three trial attempts to adapt to the test, followed by four attempts with a 30 s break between attempts to obtain their best score [34].

#### 2.3.4. Countermovement and Abalakov Jump Testing

The Countermovement Jump (CMJ) and Abalakov (ABK) tests were used to assess lower-limb power on a contact platform (Chronojump Boscosystem, Barcelona, Spain) in accordance with standardized protocols described by Bosco et al. [35].

Before testing, athletes completed a five-minute warm-up consisting of light jogging, skipping, and dynamic exercises (half-squats, lunges, and leg swings). The warm-up was standardized by duration, with exercise performed continuously for 5 min. They were familiarized with the jumping technique through four submaximal practice trials of each type (CMJ and ABK) before performing maximal attempts.

Participants completed three jumps to their preferred depth, with 60 s of rest between jumps [36]. Verbal instructions were provided, directing them to “jump as fast and as high as possible.” For the CMJ, players kept their hands on their hips, whereas for the ABK jump, they were allowed to move their arms freely. The highest value for each jump type was used for analysis [37].

#### 2.3.5. Lower-Quarter Y Balance Test

The lower-quarter Y balance test (YBT-LQ) is a functional screening tool used to assess dynamic stability in the anterior, posterolateral, and posteromedial directions. YBT-LQ has demonstrated interrater reliability with acceptable measurement error [38].

Each participant performed a lower-quarter YBT using a commercially available device (Y Balance Test, Move2Perform, Evansville, IN, USA). Before testing, right leg length was measured from the anterior superior iliac spine to the distal tip of the medial malleolus, recorded to the nearest half centimeter, using a metal tape (Cescorf, Porto Alegre, Brazil; measurement range of up to 100 cm and accuracy to 0.1 cm). Standardized verbal instructions were provided, and the researcher performed a live demonstration.

Participants completed three familiarization trials in each direction before testing. Assessment was conducted bilaterally in the anterior (ANT), posteromedial (PM), and posterolateral (PL) directions, with three valid trials per direction. All trials were performed barefoot to standardize foot–surface interaction. A trial was excluded if any of the following occurred: (i) kicking the push box, (ii) failing to return to the starting position under control, (iii) using the reach indicator for support, (iv) failing to maintain a unilateral stance on the platform, (v) touching down during testing, or (vi) placing the foot on top of the push box [39]. Reach distances were recorded to the nearest centimeter. Distances from all valid trials were recorded, and the maximum reach score in each direction was retained for analysis [40,41].

Normalized component scores for each test condition were calculated by dividing the reach distance in each direction by limb length and multiplying by 100. In addition, a composite score (COMP) was then derived as the mean of the three normalized component scores [42,43].

$$\mathrm{Composite}\mathrm{score}\;(\mathrm{COMP})=\left(\frac{\mathrm{sum}\;\mathrm{of}\;\mathrm{the}\;\mathrm{three}\;\mathrm{reach}\;\mathrm{distances}}{3\times \mathrm{limb}\;\mathrm{length}}\right)\times 100$$

To evaluate the athlete’s balance, the dynamic balance YBT protocol was used. The Y Balance Test Kit^TM^ (Move2Perform, Evansville, IN, USA) was used for evaluation. When the athletes were barefoot, they pushed the indicator (test board) with one foot in the anterior, posteromedial, and posterolateral directions. The test was performed with both feet and repeated three times for each profile [44].

#### 2.3.6. The 20 m and 30 m Sprint Tests

Participants performed three maximal 30 m linear sprints on a soccer field. Sprint times were recorded using photocell timing gates (Chronojump Bosco System, Barcelona, Spain), which recorded split times at 20 m and total sprint time at 30 m. The timing gates were positioned 1 m above the ground. The sprint was initiated following an auditory signal, with participants positioned 0.5 m behind the first photocell.

Athletes were instructed to complete the test as quickly as possible, without slowing down, until at least 5 m after passing the last photocell [36]. A passive recovery period of 2 min was provided, during which participants walked back to the starting line and then waited for the next sprint. The best performance at each distance (20 m and 30 m) was retained for analysis [45].

#### 2.3.7. T-Drill Agility Test

Time trials were recorded using photocell gates (Chronojump Boscosystem, Barcelona, Spain) placed 1 m above the ground. To start the test, the athlete began from point A, where the photocell was positioned, and sprinted toward cone B, touching it with his right hand.

The participant then moved laterally to cone C on the left and touched it with his left hand, followed by a lateral movement to cone D on the right, which was touched with the right hand. Subsequently, the athlete returned to cone B and touched it with his left hand. Finally, he sprinted back to point A, passing through the photocell to complete the test. Athletes were instructed not to cross their feet when moving laterally between cones.

**Figure 3:**
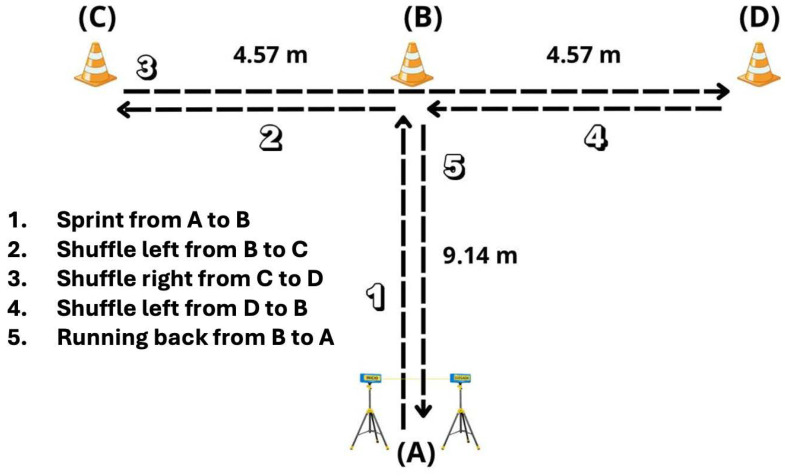
Set up of the *t*-test for agility measurement.

During the test, athletes were instructed to direct the hand toward the direction of movement; for example, when reaching for cone C while moving to the left, use the left hand. Each athlete performed the exercise twice, with a 2 min passive recovery between attempts; the fastest time was used [46]. A general description of the test is shown in Figure 3.

#### 2.3.8. Yo-Yo Intermittent Recovery Tests

The Yo-Yo Intermittent Recovery Tests (YIRT1) consisted of 2 × 20 m shuttle runs, repeated at increasing speeds, with audible beeps (13). The test began with four running bouts at 10–13 km·h^−1^, followed by seven running bouts at 13.5–14 km·h^−1^. After every eight running bouts, the speed increased by 0.5 km·h^−1^. Between runs, the players were given a 10 s active rest period in a 5 m area, during which they were encouraged to walk. The test was terminated when the players failed to complete the 2 × 20 m track within the required time on two consecutive attempts; the distance covered was recorded [47,48].

### 2.4. Statistical Analysis

All statistical analyses were performed using Jamovi statistical software (The jamovi project 2025, version 2.6; Sydney, Australia, https://www.jamovi.org). Descriptive statistics were calculated for all variables and presented as mean ± standard deviation (SD). To examine positional differences in anthropometric, body composition, and dynamic balance variables, independent sample *t*-tests were used for normally distributed variables with homogeneous variances, whereas Mann–Whitney U tests were applied for non-normally distributed variables. A Bonferroni correction was applied to avoid a Type I error. Effect sizes were reported as Cohen’s d for *t*-test and rank-biserial correlation (r) for the Mann–Whitney U test. For interpretation, we used conventional benchmarks: small effect (d ≥ 0.20), medium effect (d ≥ 0.50), and large effect (d ≥ 0.80); and r ≈ 0.10 (small), 0.30 (medium), 0.50 (large) [49].

To examine positional differences in somatotype and physical performance variables, a set of MANOVA tests using Pillai’s Trace as the multivariate statistic was conducted. Pillai’s Trace was used as the multivariate test statistic because it is considered the most robust statistic under violations of the assumptions of multivariate normality [50]. Assumptions of homogeneity of covariance matrices and normality were assessed using Box’s M and Shapiro–Wilk tests, respectively [51,52]. When appropriate, follow-up univariate ANOVAs were conducted for each dependent variable, even in the absence of a significant multivariate effect. Partial eta squared (η^2^p) was calculated as an estimate of effect size, and effect sizes were interpreted using conventional thresholds: negligible effect, η^2^p < 0.01; small effect, η^2^p = 0.01–0.05; moderate effect, η^2^p = 0.06–0.13; large effect, η^2^p ≥ 0.14 [20]. Statistical significance was set at *p* < 0.05.

## 3. Results

### Anthropometric Profile

Table 1 shows the results of the anthropometric analysis by playing position. A set of multiple independent *t*-tests and Mann–Whitney U tests was conducted, with Bonferroni correction applied to adjust for multiple testing. After adjustment, only significant positional differences were detected in the sum of six skinfolds (Σ6SKF; d = 0.90) and eight skinfolds (Σ8SKF; d = 0.94), both showing large effect sizes.

Next, positional differences in body composition were evaluated using independent-samples *t*-tests and Mann–Whitney U tests, with a Bonferroni correction applied to adjust for multiple comparisons (Table 2). After correction, significant differences were observed only in Body Fat % (t (38) = –3.11, *p* = 0.028, d = 0.98) and fat mass (U = 101.00, *p* = 0.049, r = 0.49), indicating higher adiposity in handlers, both indicating large effect sizes and higher adiposity in handlers. No significant differences were found in the rest of the variables (*p* < 0.05).

Table 3 summarizes comparisons of somatotype components and the somatotype attitudinal mean (SAM) between cutters and handlers. Before the primary analysis, we assessed the assumptions of multivariate normality and homogeneity of variances. Assumption testing indicated homogeneity of covariance matrices, as indicated by Box’s M (χ2 (10) = 9.19, *p* = 0.515). However, the results of a Shapiro–Wilk test indicated that the assumption of multivariate normality was violated (W = 0.74, *p* < 0.001).

Hence, the MANOVA test revealed a significant effect of playing position on somatotype (Pillai’s Trace = 0.29; F (4,34) = 3.49; *p* = 0.017). The univariate analysis indicated that handlers exhibited significantly greater endomorphy than cutters (F (1, 37) = 11.73; *p* = 0.002, η^2^p = 0.24), representing a large effect size and suggesting greater relative adiposity. Whereas no differences were observed in mesomorphy, ectomorphy, or SAM (*p* > 0.05, η^2^p = 0.06).

**Figure 4:**
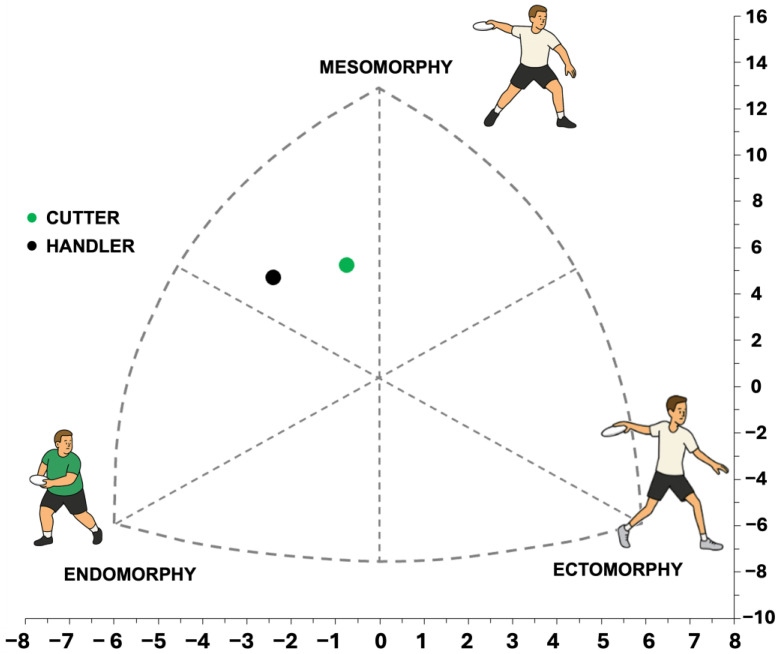
Mean somatotype distribution of cutters (n = 20) and handlers (n = 20) according to the Heath–Carter method. Each point represents the group mean somatotype plotted using standardized X–Y coordinates derived from the three-component model. The horizontal axis represents the difference between ectomorphy and endomorphy, while the vertical axis reflects mesomorphic predominance relative to the other two components. The central region reflects balanced somatotype profiles, whereas displacement toward each vertex indicates predominance of the corresponding component Heath–Carter method.

Thus, cutters exhibited an endo-mesomorph somatotype (2.99–5.20–2.24), whereas handlers also demonstrated an endo-mesomorph profile (4.15–5.31–1.74). Although handlers showed greater endomorphy, the overall somatotype configuration was similar across positions, as reflected by comparable SAM values. To facilitate interpretation, group mean somatotype coordinates are illustrated in Figure 4.

After studying the anthropometric and somatotype profiles, we conducted two MANOVAs separately to assess positional differences in physical performance variables. We again checked the assumptions of the test before analysis and found that both multivariate normality (W = 0.52, *p* < 0.001) and homogeneity of covariance matrices were violated (χ^2^ _(45)_ = 70.89, *p* = 0.008). We again used the Pillai–Bartlett test to interpret the overall results: Pillai’s Trace = 0.40; F (9,27) = 1.97; *p* = 0.083.

At the univariate level, analysis revealed that cutters outperformed handlers in vertical jump, agility, and the 20 m sprint (*p* < 0.5), along with a trend in the 30 m sprint (*p* = 0.058), as shown in Table 4. On the contrary, no positional differences emerged in HGS, upper-limb power, or Yo-Yo IR1 performance (Table 4).

Finally, multiple independent *t*-tests with a Bonferroni correction were run to examine positional differences in dynamic balance (Supplementary Table S1). The analysis revealed no significant differences in either limb or composite scores across positions (*p* > 0.05). Additionally, effect sizes were small to moderate (d = 0.09–0.52), indicating limited explained variance. Overall, these findings suggest that dynamic balance performance was similar between cutters and handlers.

## 4. Discussion

This cross-sectional study examined positional differences between cutters and handlers among male ultimate frisbee players. The main findings indicate that handlers exhibited higher body fat percentage, fat mass, and endomorphy, whereas cutters demonstrated superior sprint performance (20 m), agility, and vertical jump height (CMJ and ABK). No positional differences were observed in intermittent endurance, upper-limb power, or dynamic balance.

### 4.1. Positional-Based Differences in Anthropometric and Body Composition Profiles

Anthropometric dimensions, body composition indicators, and somatotype components are established determinants of locomotor efficiency, force production, and metabolic cost in intermittent field sports [12,33,53]. While anthropometric and somatotype profiles provide an integrated representation of structural and compositional characteristics [54], adiposity has been negatively associated with physical performance across different sports [33,37,55,56,57]. Despite limited evidence in ultimate frisbee, previous studies have described the anthropometric characteristics of well-trained players and reported values comparable to those observed in the present study [58]. However, researchers did not consider the positional roles in their analyses, making it difficult to determine whether morphological differences exist between cutters and handlers. In UF, positional roles could imply distinct physical and physiological demands that can influence long-term morphological development. Therefore, exploring positional differences in terms of anthropometric and body composition becomes relevant.

In our study, although anthropometric variables did not reach statistical significance after Bonferroni correction, effect size patterns still provide meaningful insights (Table 1). Here, handlers exhibited moderately higher skinfold values, greater waist and hip girths, and higher fat mass and body fat % (Table 2), consistent with previous reports [58]. Regarding somatotype, although both cutters and handlers were classified as endo-mesomorphs, the endomorphy component was greater in handlers (*p* < 0.01; η^2^p = 0.24), reflecting, again, a greater proportion of body fat and a softer overall body composition [59,60].

These positional differences in adiposity may reflect distinct functional demands and long-term adaptation. Cutters are typically exposed to repeated high-intensity accelerations and sprinting, which may favor lower fat mass, whereas handlers experience comparatively lower mechanical and metabolic loading. Such differences in locomotor and energetic demands may reduce the selective pressure to maintain lower fat mass, leading to meaningful variations in body composition across positions. These findings are consistent with evidence from other intermittent sports, in which positions that do not rely on rapid acceleration and repeated sprinting tend to exhibit higher endomorphy and lower leanness [55,61,62,63].

To the best of our knowledge, only one previous study has examined the somatotype profile in ultimate Frisbee players [58], reporting an endo-mesomorph profile in both handlers and cutters, comparable to that observed in our study. However, that study did not report differences within somatotype components, making our findings the first to document statistically significant differences in endomomorphy between playing roles. Despite this, neither mesomorphy nor ectomorphy differed significantly between positions, suggesting that structural build and linearity remain broadly similar across roles, while variations in adiposity may be more functionally relevant to positional demands.

### 4.2. Physical Performance

During a match, players are required to perform repeated high-intensity actions, such as CODs, sprints, accelerations, and decelerations, which directly influence tactical behaviors [4]. However, the nature and frequency of these actions vary by position. Cutters must repeatedly create space between opponents to receive passes, which requires frequent high-intensity acceleration and deceleration efforts [38,39], thereby demanding high agility and power in the lower limbs. In contrast, handlers focus on facilitating the disc’s movement across the pitch, maintaining possession, and initiating offensive actions [2] through accurate passes. These roles rely more on technical throwing proficiency, spatial awareness, and decision-making efficiency than on repeated maximal-speed actions [64].

These specific physical demands across positions have been previously documented in official matches [8], with cutters covering greater distances during high-intensity acceleration and deceleration, whereas handlers tend to cover greater distances at lower intensities. Consistent with this framework, results in the present study showed that cutters outperformed handlers in both short-distance sprinting and agility, with moderate and large effects, respectively (Table 4). These findings suggest that the cutter role is associated with greater agility and velocity, enabling them to make sudden COD to catch the disc, attack downfield spaces, and create scoring opportunities more than handlers [23,65]. Interestingly, our results are comparable to previous reports from the U-23 Colombian national team but differ from those from the Malaysian and Colombian UFP, in which no significant differences were observed in sprint or agility performance [5,66]. Such discrepancies may be partially explained by methodological differences, including sample size, a mixed sample (male and female participants), the period of the season when the tests were conducted, or reduced statistical power in those studies.

In addition to sprinting and agility performance, lower-limb power was a discriminator among playing positions. Cutters demonstrated higher jump heights in both the CMJ and the ABK jump, with moderate and large effect sizes, respectively, indicating greater stretch-shortening cycle utilization and improved neuromuscular coordination. These findings are similar to those reported by other researchers in Colombian UFP [65] and align with the functional requirements of the cutter role.

On the other hand, despite the positional differences observed in sprinting, agility, and lower-limb power, no significant differences were found between cutters and handlers in Yo-Yo IR1 performance. These results are consistent with previous reports by [65], although the absolute values observed in the present study were lower than those reported by Krustrup and Mohr (2015) [4]. Although cutters are exposed to a higher frequency of high-intensity actions during the game, this movement pattern does not necessarily translate into superior intermittent endurance capacity. Indeed, the total distance covered during matches does not appear to differ by position [8], and most of the game is spent in low- and moderate-intensity zones [7]. Additionally, the Yo-Yo IR1 primarily assesses the player’s ability to recover from intermittent high-intensity actions rather than from position-specific sprinting or acceleration demands. Consequently, although cutters exhibit positional specialization in sprinting, agility, and lower-limb power, intermittent endurance aptitude appears represent a shared physical capacity rather than a discriminating factor among playing positions.

Finally, no positional differences were observed in HGS and upper-limb power (Table 4) or in dynamic balance performance (Supplementary Table S1). These findings indicate that upper-limb power production and general postural control are shared physical requirements in UFP rather than position-specific attributes. This may reflect that, during the game, both the cutter and the handler repeatedly perform various throwing techniques (Backhand, Forehand, Hammer) and catching techniques (one- or two-handed), while stabilizing their bodies under defensive pressure [24,67]. Therefore, shared playing patterns may explain the homogeneous profiles observed in these variables. Moreover, throwing performance in ultimate can be influenced mainly by technical proficiency and coordination rather than maximal upper-limb power [19].

Thus, these results suggest that physical performance profiles in ultimate may be shaped by position-specific game demands, particularly in agility, velocity, and lower-limb power, as observed in other invasion sports [68,69,70,71]. Collectively, these observations underscore the relevance of position-specific profiling in understanding performance variability within UF. Accordingly, the present study provides a comprehensive positional characterization of male UFP by integrating anthropometric, body composition, somatotype, and physical performance assessments using standardized field-based protocols, thereby contributing novel evidence in an understudied sport context.

Some limitations should be considered when interpreting these findings. The use of a convenience sample may limit generalizability to other competitive levels or populations. Although the sample size was sufficient to detect large between-position effects, statistical power may have been limited for smaller or moderate differences, and these effects should therefore be interpreted with caution. A further limitation of this study is that hydration status was not objectively monitored prior to testing. Although participants were instructed to maintain habitual hydration and avoid stimulant beverages, variations in hydration status may have influenced performance outcomes, particularly in high-intensity intermittent tasks [72,73]. In addition, while the field-based tests employed are standardized and widely used in sport science, they are not specific to UF. Future research should seek to validate performance assessments that better capture the technical and tactical demands unique to this sport.

## 5. Conclusions

This study provides a comprehensive positional comparison of anthropometric characteristics, body composition, somatotype, and physical performance in male UFP. Although general anthropometric dimensions were similar between cutters and handlers, meaningful positional differences emerged in body composition and neuromuscular performance. Specifically, handlers had higher fat mass percentage and greater endomorphy, whereas cutters showed superior sprinting speed, agility, and vertical jump performance, reflecting greater lower-limb neuromuscular demands related to their tactical role.

In contrast, intermittent endurance capacity, upper-limb power, and dynamic balance did not show large positional differences, suggesting that these attributes reflect shared physical requirements inherent to the sport rather than position-specific characteristics. Collectively, these findings indicate that positional specialization in UF is primarily expressed through lower-limb speed, agility, and vertical jump rather than through intermittent endurance or upper-body performance.

These findings have practical implications for position-specific training strategies that prioritize neuromuscular development for cutters while maintaining global conditioning capacities across all players. Additionally, coaches may emphasize sprint mechanics, COD ability, and lower-limb power in cutters, reflecting their greater exposure to high-intensity off-the-disc movements. Furthermore, anthropometric and somatotype profiling may support talent identification and position allocation by aligning individual morphological characteristics with the specific physical demands of each role.

## Figures and Tables

**Table 1:** Anthropometric profile of Ultimate Frisbee Players.

Variable	Cutters	Handlers	Total	Effect Size
Basic				
Height (cm)	176.0 ± 6.60	175 ± 5.07	175.50 ± 5.83	d = 0.17 ^a^
Sitting height (cm)	91.45 ± 2.86	92.10 ± 2.14	91.78 ± 2.52	d = 0.26 ^a^
Wingspan (cm)	179.30 ± 8.20	176.09 ± 6.49	177.70 ± 7.48	d = 0.43 ^a^
Weight (kg)	74.03 ± 9.33	76.66 ± 6.08	75.35 ± 7.88	d = 0.33
BMI (kg/m^2^)	23.99 ± 2.80	25.19 ± 2.52	24.59 ± 2.70	d = 0.45 ^a^
Waist-to-Hip ratio	0.81 ± 0.03	0.83 ± 0.04	0.82 ± 0.04	d = 0.37 ^a^
Girths (cm)				
Arm (relaxed)	29.55 ± 2.63	30.82 ± 2.56	30.19 ± 2.64	d = 0.49 ^a^
Arm (flexed and tensed)	32.15 ± 2.69	32.50 ± 2.84	32.33 ± 2.74	r = 0.09 ^b^
Waist	78.42 ± 5.06	82.53 ± 5.27	80.47 ± 5.51	d = 0.79 ^a^
Hip	96.53 ± 6.39	99.85 ± 4.44	98.19 ± 5.68	d = 0.60 ^a^
Calf	36.87 ± 2.73	37.23 ± 1.63	37.05 ± 2.21	d = 0.16 ^a^
Skinfolds (mm)				
Triceps	8.36 ± 3.62	11.35 ± 3.64	9.86 ± 3.89	r = 0.47 ^b^
Subscapular	9.98 ± 4.26	12.78 ± 3.56	1.38 ± 4.13	r = 0.51 ^b^
Biceps	4.10 ± 1.25	5.83 ± 2.36	4.96 ± 2.06	r = 0.53 ^b^
Supraspinal	6.97 ± 3.28	10.20 ± 3.79	8.59 ± 3.86	r = 0.56 ^b^
Abdominal	15.00 ± 6.68	21.50 ± 6.18	18.25 ± 7.15	d = 1.01 ^a^
Iliac crest	12.36 ± 5.91	17.98 ± 6.82	15.17 ± 6.91	d = 0.88 ^a^
Front thigh	10.44 ± 4.44	12.28 ± 4.33	11.36 ± 4.43	r = 0.28 ^b^
Medial calf	6.38 ± 3.22	7.70 ± 2.38	7.06 ± 2.88	r = 0.42 ^b^
Σ6SKF	57.13 ± 21.91 *	75.80 ± 19.58	66.47 ± 22.59	d = 0.90 ^a^
Σ8SKF	73.60 ± 28.30 *	99.6 ± 26.85	86.60 ± 30.24	d = 0.94 ^a^
Lengths (cm)				
Arm length	34.02 ± 1.79	33.90 ± 1.49	33.96 ± 1.63	d = 0.07 ^a^
Forearm length	27.38 ± 1.41	26.60 ± 1.44	26.99 ± 1.46	d = 0.54 ^a^
Hand length	19.61 ± 1.06	19.38 ± 1.02	19.50 ± 1.04	d = 0.23 ^a^
First–fifth finger distance	21.32 ± 1.28	20.93 ± 1.28	21.13 ± 1.28	d = 0.31 ^a^
Arm length (left)	33.83 ± 1.94	33.88 ± 1.24	33.85 ± 1.61	d = −0.03 ^a^
Forearm length (left)	26.73 ± 1.55	26.35 ± 1.35	26.54 ± 1.45	d = 0.27 ^a^
Hand length (left)	19.68 ± 1.02	19.32 ± 0.99	19.50 ± 1.01	d = 0.35 ^a^
First–fifth finger distance (left)	21.89 ± 2.02	21.02 ± 1.09	21.46 ± 1.66	r = 0.30 ^b^
Bone breadth (cm)				
Humerus	7.09 ± 0.29	7.08 ± 0.33	7.08 ± 0.31	r = 0.07 ^b^
Femur	10.02 ± 0.45	9.82 ± 0.40	9.92 ± 0.43	r = 0.18 ^b^
Bystyloid	6.01 ± 0.74	5.88 ± 0.34	5.95 ± 0.57	r = 0.02 ^b^
Hand	8.64 ± 0.39	8.52 ± 0.54	8.58 ± 0.47	r = 0.19 ^b^
Humerus (left)	7.00 ± 0.40	7.17 ± 0.80	7.08 ± 0.63	r = 0.08 ^b^
Bystyloid (left)	5.92 ± 0.30	5.88 ± 0.35	5.90 ± 0.32	r = 0.03 ^b^
Hand (left)	8.55 ± 0.50	8.44 ± 0.41	8.49 ± 0.45	r = 0.22 ^b^

Σ6SKF = sum of six skinfolds (biceps, triceps, subscapular, abdominal, iliac crest, and supraspinal folds); Σ8SKF = sum of six skinfolds (biceps, triceps, subscapular, abdominal, suprailiac, supraspinal folds, front thigh and medial calf). ^a^ Parametric comparisons were performed using independent samples *t*-tests (effect size reported as Cohen’s d). ^b^ Non-parametric comparisons were performed using the Mann–Whitney U test (effect size reported as r). * Denotes *p* < 0.05 vs. handlers.

**Table 2:** Body composition variables in Ultimate Frisbee players.

Variable	Cutters	Handlers	Total	Effect Size
Body Fat % (Faulkner)	11.95 ± 2.45 *	14.32 ± 2.37	13.14 ± 2.67	d = 0.98 ^a^
Fat mass (kg)	8.95 ± 2.67 *	11.08 ± 2.51	10.02 ± 2.78	r = 0.49 ^b^
Bone mass (kg)	12.63 ± 1.75	12.15 ± 0.98	12.39 ± 1.42	r = 0.07 ^b^
Residual mass (kg	17.84 ± 2.25	18.48 ± 1.46	18.16 ± 1.90	d = 0.33 ^a^
Muscle mass (kg)	34.60 ± 4.27	34.96 ± 2.76	34.78 ± 3.55	d = 0.10 ^a^
Upper arm muscle area (cm^2^)	57.99 ± 8.95	59.56 ± 10.67	58.78 ± 9.76	r = 0.10 ^b^
Upper arm muscle area (cm^2^, left)	53.65 ± 10.19	57.42 ± 10.44	55.54 ± 10.36	d = 0.37 ^a^

All data are presented as mean ± standard deviation. ^a^ Parametric comparisons were performed using independent samples *t*-tests (effect size reported as Cohen’s d). ^b^ Non-parametric comparisons were performed using the Mann–Whitney U test (effect size reported as r). * Denotes *p* < 0.05 vs. handlers.

**Table 3:** Somatotype characteristics of Ultimate Frisbee players.

Variable	Cutters	Handlers	Total	F	η^2^p
Endomorphy	2.99 ± 1.30 **	4.15 ± 1.22	3.57 ± 1.38	11.73	0.24
Mesomorphy	5.20 ± 1.25	5.31 ± 1.17	5.26 ± 1.19	0.07	0.00
Ectomorphy	2.24 ± 1.26	1.74 ± 1.13	1.99 ± 1.20	2.38	0.06
SAM	1.88 ± 1.47	1.89 ± 0.63	1.88 ± 1.11	0.37	0.01

All data are presented as mean ± standard deviation. Positional differences were examined using univariate ANOVAs following the multivariate analysis. Effect size reported: partial eta squared (η^2^p) ** denotes *p* < 0.01 vs. handlers.

**Table 4:** Physical performance variables in cutters and Handlers.

Variable	Cutters	Handlers	Total	F	η^2^p
DHGS (kg)	42.37 ± 7.18	41.50 ± 4.24	41.92 ± 5.79	0.57	0.02
NDHGS (kg)	42.52 ± 8.13	38.91 ± 5.04	40.66 ± 6.88	3.09	0.08
SMBT (m)	4.07 ± 0.50	3.87 ± 0.58	3.97 ± 0.54	0.55	0.02
CMJ (cm)	40.93 ± 6.54 *	36.38 ± 4.71	38.53 ± 5.03	5.91	0.14
Abalakov (cm)	46.39 ± 7.88 **	40.20 ± 4.68	47.13 ± 7.04	9.71	0.22
Agility test (s)	10.16 ± 0.69 *	10.69 ± 0.61	10.44 ± 0.70	6.44	0.16
20 m sprint (s)	3.11 ± 0.17 *	3.21 ± 0.15	3.16 ± 0.17	4.48	0.12
30 m sprint (s)	4.33 ± 0.41	4.47 ± 0.21	4.41 ± 0.33	3.86	0.10
Yo-Yo IR1 (m)	982.22 ± 353.60	940.00 ± 347.62	960.00 ± 346.35	0.11	0.00

All data are presented as mean ± standard deviation. */** denotes *p* < 0.05/*p* < 0.01 vs. handlers. Positional differences were examined using univariate ANOVAs following the multivariate analysis. DHGS: dominant handgrip strength side; NDHGS: non-dominant handgrip strength; SMBT: seated medicine ball throw test; CMJ: countermovement jump: Yo-Yo IR1: intermittent recovery test level 1; η^2^p: Partial eta squared.

## Data Availability

The data presented in this study are available on request from the corresponding author. The data are not publicly available due to privacy.

## Supplementary Materials

The following supporting information can be downloaded at: https://www.mdpi.com/article/10.3390/jfmk11010128/s1, Table S1. Descriptive analysis of the relative reach score during Y-Balance Test–Lower Quarter (YBT-LQ) in Ultimate Frisbee players (n = 40).
