# Supplementary material for: Beyond the Disc: Positional Differences in Morphological and Physical Performance Characteristics Among Male Ultimate Frisbee Players
Source: J Funct Morphol Kinesiol. 2026 Mar 22;11(1):128. doi: 10.3390/jfmk11010128 (PMC13027780; doi:10.3390/jfmk11010128)
Supplement: Supplementary file 1 [file jfmk-11-00128-s001.zip › jfmk-4107206-supplementary.pdf]

Supplementary Materials

# Beyond the Disc: Positional Differences in Morphological and Physical Performance Characteristics Among Male Ultimate Frisbee Players

**Cristian Hernández [1], María Alejandra Camacho-Villa [2,3], Nuria Sánchez-Hernández [4], Luis Gabriel Rangel Caballero [5], Jorge Gómez-Camacho [1], Juan Carlos Saavedra [6], Jorge Enrique Buitrago-Espitia [7] and Adrián De la Rosa [7,8,*]**

[1] Programa de Maestría en Ciencias de Deporte, Universidad Santo Tomás, Bucaramanga 681011, Colombia
[2] Pain Study Group (GED), Physical Therapy School, Universidad Industrial de Santander, Bucaramanga 680002, Colombia
[3] Performance and Health Group, Department of Physical Education and Sport, Faculty of Sports Sciences and Physical Education, University of A Coruna, 15001 A Coruña, Spain
[4] Physical Education and Sports Department, University of Valencia, 46010 Valencia, Spain
[5] Grupo Ser, Cultura y Movimiento, Facultad de Cultura Física Deporte y Recreación, Universidad Santo Tomás, Bucaramanga 681011, Colombia
[6] Physical Activity and Sport Program, Unidades Tecnológicas, Bucaramanga 680006, Colombia
[7] Body, Physical Activity and Sport Study Group (GECAFD), Sports Department, Universidad Industrial de Santander, Bucaramanga 680002, Colombia
[8] Freshage Research Group, Department of Physiology, Faculty of Medicine, University of Valencia, CIBERFES, Fundación Investigación Hospital Clínico Universitario/INCLIVA, 46010 Valencia, Spain
* Correspondence: adrian.rosa@uv.es

**Table S1. Descriptive analysis of the relative reach score during Y- Balance Test – Lower Quarter (YBT-LQ) in Ultimate Frisbee players (n=40)**

| Side | Anterior Reach (%LL) (n=40) | | | | Posteromedial Reach (%LL) (n=40) | | | | Posterolateral Reach (%LL) (n=40) | | | | Composite Score (%LL) (n=40) | | | |
|---|---|---|---|---|---|---|---|---|---|---|---|---|---|---|---|---|
| | Cutters | Handlers | Total | ES | Cutters | Handlers | Total | ES | Cutters | Handlers | Total | ES | Cutters | Handlers | Total | ES |
| DS | 66.18±7.34 | 62.45±7.09 | 64.27±7.36 | 0.52 | 117.13±6.82 | 115.20±8.98 | 116.14±7.96 | 0.24 | 112.40±9.35 | 113.28±9.23 | 112.85±9.18 | 0.09 | 98.57±6.95 | 96.98±6.90 | 97.75±6.88 | 0.23 |
| NDS | 66.98±7.21 | 62.49±8.76 | 64.67±8.26 | 0.56 | 117.86±7.16 | 116.14±8.62 | 116.98±7.89 | 0.22 | 111.97±7.25 | 112.94±8.48 | 112.46±7.82 | 0.12 | 98.93±6.05 | 97.19±6.77 | 98.04±6.40 | 0.27 |

All data are presented as mean ± standard deviation. Each reach score was normalized by lower-limb length. DS: dominant side; NDS: non-dominant side. LL%: percentage of lower limb. ES: effect size (Cohen´s d).
